# Looking to the Future of the Role of Macrophages and Extracellular Vesicles in Neuroinflammation in ALS

**DOI:** 10.3390/ijms241411251

**Published:** 2023-07-08

**Authors:** Elisabetta Carata, Marco Muci, Simona Di Giulio, Stefania Mariano, Elisa Panzarini

**Affiliations:** Department of Biological Sciences and Technologies (Di.S.Te.B.A.), University of Salento, 73100 Lecce, Italy; elisabetta.carata@unisalento.it (E.C.); marco.muci@unisalento.it (M.M.); simona.digiulio@unisalento.it (S.D.G.)

**Keywords:** macrophages, extracellular vesicles, amyotrophic lateral sclerosis, neuroinflammation, motor neuron–glial cells–macrophages communication, macrophages polarization

## Abstract

Neuroinflammation is a common pathological feature of amyotrophic lateral sclerosis (ALS). Although scientific evidence to date does not allow defining neuroinflammation as an ALS trigger, its role in exacerbating motor neuron (MNs) degeneration and disease progression is attracting research interest. Activated CNS (Central Nervous System) glial cells, proinflammatory peripheral and infiltrated T lymphocytes and monocytes/macrophages, as well as the immunoreactive molecules they release, represent the active players for the role of immune dysregulation enhancing neuroinflammation. The crosstalk between the peripheral and CNS immune cells significantly correlates with the survival of ALS patients since the modification of peripheral macrophages can downregulate inflammation at the periphery along the nerves and in the CNS. As putative vehicles for misfolded protein and inflammatory mediators between cells, extracellular vesicles (EVs) have also drawn particular attention in the field of ALS. Both CNS and peripheral immune cells release EVs, which are able to modulate the behavior of neighboring recipient cells; unfortunately, the mechanisms involved in EVs-mediated communication in neuroinflammation remain unclear. This review aims to synthesize the current literature regarding EV-mediated cell-to-cell communication in the brain under ALS, with a particular point of view on the role of peripheral macrophages in responding to inflammation to understand the biological process and exploit it for ALS management.

## 1. Introduction

Neuroinflammation is defined as the activation of the brain’s innate immune system in response to an inflammatory challenge and it is characterized by a host of cellular and molecular changes within the brain. Neuroinflammation relies on the activation of glial cells and complement-mediated pathways, the synthesis of inflammation mediators, and the recruitment of leukocytes [1,2]. If the inflammatory response becomes excessive, an increase in toxic cytokines, proteases, and free radicals levels along with the activation of glial cells occur, causing a secondary inflammatory injury [3,4]. This exacerbated inflammatory process is involved in triggering or exacerbating different neurological pathologies. Recent evidence suggests a potential role of inflammatory mechanisms also in neurological conditions not usually categorized as inflammatory, including amyotrophic lateral sclerosis (ALS) [5].

The degeneration and subsequent loss of upper and lower motor neurons (MNs) in the brain and spinal cord characterizes ALS and leads to chronic and progressive muscle weakness, and the loss of all muscle control, causing paralysis that affects patients’ quality of life and frequently results in death [6]. The vast majority of ALS cases are sporadic, and only about 10% of cases are familial due to genetic mutations in more than 30 genes that are able to cause toxic aggregates and interfere with mitochondrial functions and autophagy; degeneration mechanisms remain still unknown [7]. Superoxide dismutase 1 (*SOD1*), fused in sarcoma (*FUS*), chromosome 9 open reading frame 72 (*C9orf72*), optineurin (*OPTN*), TANK-binding kinase 1 (*TBK1*), and TAR DNA-binding protein 43 (*TDP-43*) are the most common mutated genetic forms in familial ALS cases, and different gene mutations can result in the same clinical phenotype, while diverse clinical phenotypes can result from the same mutant gene [8]. An incomplete understanding of the pathological basis of ALS determines the failure of therapeutic strategies that are based, now, only on two approved drugs, riluzole and edavarone, which are able to alleviate symptoms or delay their progression.

The recognition of the potential role of neuroinflammation in ALS is promoting several investigations that indicate that the inflammatory factors correlate with neuronal loss, even during the pre-symptomatic phase of ALS, as well as disease severity [9]. More and more pieces of evidence sustain the idea that neuroinflammation participates in causing the heterogeneity characterizing ALS [10]. Activated central nervous system (CNS) microglia and astroglia, proinflammatory peripheral lymphocytes, and macrophages have been observed in studies involving both ALS patients and transgenic mice. Also, these studies suggest a dual role for inflammation, i.e., an initial anti-inflammatory protective response, followed by a subsequent pro-inflammatory toxic response [11]. This early versus late inflammatory response could represent a potential therapeutic target [12]. However, when the proinflammatory response does not yield, the constant presence and continued production of inflammatory cytokines and reactive oxygen species can lead to cell death and further tissue damage [13].

The modulation of inflammation is based on the capability of microglia and monocytes/macrophages to display two opposite types, M1 phenotype and M2 phenotype, that can produce either cytotoxic or neuroprotective effects [14]. The dynamic shift of M1/M2 phenotypes is critically associated with neurodegenerative diseases, including ALS [15]. The changes in inflammatory cell phenotypes depend on the disease stages and severity; moreover, mastering the stage-specific switching of M1/M2 phenotypes within appropriate time windows may provide better therapeutic benefit.

Accumulating evidence indicates that neuroinflammation in ALS is systemic and a chronic pro-inflammatory microenvironment occurs both in the peripheral and central nervous system [16]. Furthermore, researchers concord that the continuous interaction and communication between these two immune systems is important in defining ALS pathology; unfortunately, this is still not well-defined.

Considerable effort has been devoted to understanding the mechanisms by which cells communicate with each other in determining inflammatory status. Many studies have revealed, in great molecular detail, processes such as transcellular signaling through chemical molecules. In recent years, signal transmission through extracellular vesicles (EVs) has emerged as a new aspect of cell-to-cell signaling [17].

Several studies report the involvement of EVs in pathological intercellular communication in a panoply of diseases, including metabolic [18], cancer [19], cardiovascular [20], etc. EVs have also gained a growing interest in neurological diseases, including cancer and neurodegeneration, as carriers of pivotal molecules representing a novel axis of intercellular communication between all cell types within CNS. Carried molecules, mainly miRNAs, can promote or inhibit the polarization of inflammation-involved cells, including microglia and macrophages, to M1 or M2 phenotypes, thus influencing the inflammatory state. Moreover, due to the capability to cross the blood–brain barrier (BBB) in both directions, from the brain to the bloodstream and vice versa, EVs released by brain cells cause the recruitment of macrophages into the brain exacerbating pathological status [21]. On the other hand, there is evidence about the impairment of the BBB in both ALS rodent models and patients leading to promote interaction between CNS and peripheral immune cells, that in turn exacerbates the neuroinflammatory responses and the severity of neurodegeneration due to the death of MNs, the damage of MNs axons and the dysfunction of neuromuscular junctions (NMJs) [22,23,24]. If the role of the activated CNS microglia and astroglia is known, the role of peripheral immunity and its interaction with CNS immunity, along with the role of EVs in this crosstalk, is still at infancy.

The possibility to detect EVs in biological fluids, including blood, trigger the growing interest in considering EVs as potential biomarkers because they reflect molecular events occurring in the brain, thereby serving as a brain liquid biopsy and a tool to develop a therapeutic personalized approach. Therefore, EVs can represent a clinical tool with wide applications in diagnosis, prognosis, and therapeutics.

Here, we focused on the current literature, showing that the EVs/macrophages axis is a key feature in the neuroinflammation exacerbating ALS pathogenesis and can be considered a new hope in therapy. It is well-recognized that the impact of peripheral monocytes/macrophages on ALS can be considered crucial as nerve macrophages were affected before microglia, so the involvement of the peripheral macrophages in the disease was likely. Moreover, the role of EVs in recruiting macrophages into CNS or mediating inflammatory status is becoming a very attractive field of research.

## 2. Immune Response Regulation and Dysregulation in the Pathogenesis of ALS

A complex interaction between genetic and environmental factors ignites pathophysiological processes underlying ALS characterized by MNs death occurring upon CNS glia and peripheral innate and adaptive immune responses dysregulation. This feature has been observed in the 10% of patients with a positive family history of ALS and in the 90% of patients with sporadic ALS without a positive family history [25]. In addition, the activation of CNS reactive microglia and astroglia and the infiltration of activated peripheral monocytes and lymphocytes have been reported in patients with sporadic ALS whose initiation is still poorly understood.

Mutations in several genes, reported in Table 1, which are able to compromise CNS glial protective responses and promote proinflammatory-mediated motoneuron injury, have been studied in clinical studies (superoxide dismutase 1-*SOD1*, TAR DNA-binding protein 43-*TARDBP*, and chromosome 9 open reading frame 72-*C9orf72*) or transgenic rodent models (OPTN (coding for optineurin (OPTN) protein), TBK1 (coding for serine–threonine protein kinase-binding kinase 1), SQSTM1 (coding for sequestosome-1 protein, also known as ubiquitin-binding protein p62), TNIP1 (coding for tumor necrosis factor α-induced protein 3 interacting protein 1), VPC (coding for valosin-containing protein), and CX3CR1 (coding for chemokine receptor 1, also known as fractalkine receptor). The mutated proteins encoded by these genes lead to ALS and promote immune dysfunction, representing the evidence that immune system-driven inflammatory mechanisms contribute to the pathogenesis of ALS. Moreover, the mutation of these genes indicates the contribution of autophagy (i.e., the intracellular process that allows the sequestering and orderly degradation and recycling of aggregated misfolded proteins and dysfunctional cellular organelles), nuclear factor-kappa B (NF-κB), and the downstream nucleotide-binding domain-like receptor protein 3 (NLRP3) inflammasome (i.e., a multiprotein complex responsible for the activation of inflammatory responses that promotes the maturation and secretion of proinflammatory cytokines interleukin IL-1β and IL-18) to the pathogenesis of ALS [10].

Whilst not the focus of this review, below are reported the involvement of the main genes affecting inflammatory status in ALS.

*OPTN* was first identified as an ALS causative gene by a loss-of-function mutation in 2010, and more than 20 mutations are known. Due to numerous possible interacting partners, optineurin is involved in several cellular processes, including autophagy, vesicular trafficking, Golgi maintenance, secretion, receptor recycling, and the negative regulation of the NF-κB pathway [28]. The transcription factors NF-κB family are the key regulators of cytokine production; it is ubiquitously expressed in mammalian cells, including neurons, and it can protect neurons, regulate neuroinflammation and contribute to neurodegeneration.

Whether and how *OPTN* affects the NF-κB pathway in ALS is still a controversial issue: *OPTN* normally suppresses NF-κB activity, and its absence or mutation causes NF-κB translocation to the nucleus and expression of proinflammatory genes involved in neurodegeneration in microglia, neurons, oligodendrocytes and astrocytes [28,29,30,31]. Both sALS and fALS patients, bearing optineurin-depleted NF-κB activity in microglia, which increases neuronal necroptosis due to a severe axonopathy, are able to induce oligodendrocyte death and the degeneration and swelling of motor neuron axons. Similar findings are observed in the spinal cord of ALS patients [30]. These data suggest that oligodendrocytes and microglia contribute to optineurin-mediated neurodegeneration.

*TBK1* mutations, including nonsense, frameshift, missense, and deletion, have been observed in both sporadic and familial ALS patients. The TBK1 protein is involved in: innate immunity signaling by the induction of type-1 interferons; in autophagy and mitophagy by impairing the phosphorylation of autophagy adaptors; in efficient cargo recruitment in autophagosomes as well as autophagosomes maturation via the disruption of microtubule dynamics and the consequent inhibition of fusion with lysosomes; and, finally, in the accumulation of defective mitochondria [32]. The involvement of TBK1 mutations in neuroinflammation during ALS relies on the removal of T cells protective regulation (due to the ability of CD4+ cells in stabilizing microglial activation, decreasing pro-inflammatory cytokines, and increasing growth factor IGF-1) observed in both the spinal cord lesions of ALS patients and *TBK1* knockout mice, leading to a decrease in T cell number in the CNS [33].

Since 1993, the most frequent mutations associated with ALS are those in the SOD1 gene encoding the antioxidant enzyme Cu/Zn superoxide dismutase. The pathogenicity of SOD1 mutations is due to the accumulation of its misfolded aggregates. SOD1 ignites an inflammation process by controlling the activity of caspase-1, which is able to cleave and, in turn, activate IL-1β pro-inflammatory cytokine. ALS patients show elevated levels of caspase-1 and inflammation due to accumulation inside the neurons of misfolded SOD1 enzymes that gradually destroy the motor neurons. Studies using mice carrying mutant SOD1 demonstrate that through blocking interleukin-1ß, the inflammatory reaction is attenuated, and the animals’ life spans are prolonged along with a reduction in symptoms [34].

### 2.1. Interactions between Glial Cells Are Key Contributors to ALS Neuroinflammation Status

Neuronal and glial cells, including microglia, oligodendrocytes, and astrocytes, interact for proper brain development and function. The activation of glial cells differs between ALS models: in SOD1 mouse models, extensive glial cell activation occurs, in contrast with that observed in TBK1, OPTN, and TDP-43 models [35,36,37]. In particular, in transgenic TDP-43 mice, microglia activation is inhibited by damaged neurons since the stop in TDP-43 expression elicits the activation of microglia with the consequent cleaning of aggregated TDP-43 from surviving neurons. Likewise, microglia activation in ALS patients strictly depends on mutated genes: patients carrying SOD1 mutations had a higher microglial activation than C9orf72 ones [37].

### 2.2. Microglia

The primary immune cells of the CNS are microglia. They derive from primitive macrophages located in the mesoderm and invade CNS during its development [38]. Microglia are highly motile cells by continuously extending and retracting their processes to constantly survey the micro-environment to protect neurons from infection or injury by phagocytosing dead cells and debris [39,40]. The activation of microglia involves graded and temporal changes in their morphology and gene expression [41,42], proliferation, and the release of a wide variety of cytokines that, in turn, have protective or detrimental effects on neurons [43]. Two extremes in microglia phenotypes exist in response to different stimuli: an M1 or classically activated phenotype, able to produce reactive oxygen species (ROS) and proinflammatory cytokines toxic to neurons, and an M2 or alternatively activated phenotype that, by producing anti-inflammatory cytokines as well as neurotrophic factors, is protective to neurons. The uptake of apoptotic cells or exposure to anti-inflammatory cytokines, such as IL-10 and transforming growth factor-β, induces in microglia another M2 anti-inflammatory phenotype, known as the acquired deactivation phenotype [15]. However, this categorization of microglial states is very simplified; in fact, the terminology of M1 and M2 derives from peripheral macrophages characterized by a phenotypic switch, and conversely, microglia exhibit a lower grade of plasticity [44]. It is now clear that microglia elicit graded and context-dependent responses when activated and play a pro-inflammatory or anti-inflammatory role in response to surrounding cells [45,46].

In ALS mice, it has been observed that during the progression of the disease, resident microglia increase their number, and their activation status is a continuum between the neuroprotective M2 *vs.* toxic M1 phenotypes [47,48]. In ALS mouse models, microglia are initially neuroprotective in the early stages of the disease and only subsequently transit to a pro-inflammatory state [47].

The occurrence of four different phenotypes of microglial cells, based on their morphology, has been described in SOD1G93A transgenic mice, a model of ALS: type S (surveillant microglia) and types R1, R2, and R3 (reactive microglia). Type S microglia display the typical ramified morphology of surveillant microglia, and were mainly present in wild-type controls; type R1 microglia with small cell bodies and shorter and simpler processes are seen at the early stage of disease in SOD1G93A mice; type R2 microglia morphologically similar to type R1 transiently occur in the middle stage of disease; type R3 microglia exhibit a bushy shape, and are typical at the end stage of disease [49]. Consistently, microglia exhibit an anti-inflammatory profile with attenuated TLR2 responses to a controlled immune challenge and an overexpression of anti-inflammatory IL-10 at the pre-onset phase of SOD1-mediated disease [50]; conversely, a pro-inflammatory phenotype with high levels of NOX2, the subunit of nicotinamide–adenine–dinucleotide–phosphate oxidase, is prevalent in the end-stage phase of SOD1-mediated disease [51]. The specific molecular determinants of the inflammatory status transition are far from being clearly understood but appear to derive from communications between the motor neuron projections outside the blood–brain barrier at the neuromuscular junction and the peripheral immune cells. Primary MNs prepared from spinal cords of C57BL/6 mice at embryonic day 13–14 mutated in the TDP-43 gene are able to activate microglia and upregulate the release of NOX2, TNF-α, and IL-1β pro-inflammatory mediators and elicit a more toxic microglial phenotype [52]. mSOD1-expressing microglia underlie phenotypic transformation during the disease. mSOD1 microglia isolated from ALS mice at disease onset express higher levels of markers of M2 and lower levels of markers of M1 compared to mSOD1 microglia isolated from ALS mice at end-stage disease. Moreover, when co-cultured with wild-type MNs, these mSOD1 M2 microglia are neuroprotective and enhance motoneuron survival; conversely, end-stage mSOD1 M1 microglia are toxic and increase motoneurons death [47]. Several findings suggest that during ALS progression, the transition from M2 is more than the transition to M1. For example, SOD1G93A microglia overexpress, both in the pre-symptomatic stage and in end-stage disease, the components of inflammation, such as insulin growth factor-1 (IGF-1), whose release is suppressed in a pro-inflammatory (M1) environment and enhanced in an M2 anti-inflammatory environment [53]. Furthermore, an analysis of transcriptome changes of SOD1G93A microglia suggests that the activation of genes involved in anti-inflammatory pathways, including, Igf1, progranulin, and Trem2, coexists with the upregulation of genes coding for neurotoxic factors, such as matrix metalloproteinase-12 and classical proinflammatory cytokines, without a significant prevalence of M1 or M2 phenotypes at any time point during disease progression [48].

On this basis, the possibility of appropriately modulating microglial phenotypes, enhancing the anti-inflammatory properties, and inhibiting or reducing M1 toxicity, could be a promising therapeutic strategy for ALS.

### 2.3. Oligodendrocytes

Oligodendrocytes provide metabolic support to neurons and maintain the myelin sheath required for the neuronal saltatory conduction of action potentials. In mSOD1 mice, a degeneration of oligodendrocytes accompanied by microglial activation occurs, and the removal of mSOD1 from oligodendrocytes results in a delay in disease onset and microglial activation. Oligodendrocyte progenitor cells (OPCs) replace lost oligodendrocytes by a differentiation mechanism influenced by microglial reactive state [54]. However, the relationship between microglia and OPCs in the context of ALS is still not well-understood.

### 2.4. Astrocytes

Astrocytes are the most abundant glial cells in the CNS and are involved in metabolic homeostasis, synapse regulation, and immune functions, providing structural and trophic support to neurons and maintaining the BBB integrity [55]. Even if astrocytes are not immune cells per se, they can respond to cytokines released by microglia, proliferate and secrete cytokines and, thus, they initiate communication with microglia and influence the neuroinflammation [56,57].

The mechanisms initiating and regulating the interplay between microglia and astrocytes in ALS have been partially addressed in the mSOD1 mouse model and they are still not well-understood. Boille demonstrated that by reducing the mSOD1 levels in microglia, disease progression sharply slows even if no effects have been observed in the early disease phase [58]. In the same manner, using mSOD1G37R mice, a diminished expression of the mSOD1 levels in astrocytes does not affect the onset, but microglial activation and disease progression slow [59]. Lepore and coworkers demonstrated that the transplantation of wild-type astrocyte precursors into the mSOD1 mouse spinal cord reduces microglial activation [60], and transplantation into the wild-type mice of SOD1G93A glial-restricted precursor cells differentiated efficiently into astrocytes and induced not only microglial activation, but also MNs death and forelimb motor and respiratory dysfunction [61]. Most likely, the upregulated transforming growth factor-β1 (TGF-β1) in murine and human ALS astrocytes regulates the inflammatory response of microglia as demonstrated in a study of Endo, reporting that the astrocyte-specific overproduction of TGF-β1 in SOD1(G93A) mice accelerates disease progression via IGF-I production in deactivated microglia. This result is corroborated using the pharmacological administration of a TGF-β signaling inhibitor after disease onset, which is able to extend the survival time of SOD1(G93A) mice [62].

### 2.5. Can Peripheral Immune Cells Contribute to ALS Neuroinflammation Status?

There is mounting evidence that several other circulating innate immune cell subsets, including macrophages, monocytes, dendritic cells (DC), natural killer (NK) cells, mastocytes, and neutrophils, could contribute to ALS progression in transgenic rodent models of the disease. Even if the peripheral immune components are not capable to infiltrate into the CNS and CNS is consequently considered to be immunologically privileged, there is evidence that peripheral immune cells actively communicate with the CNS immune ones. Data from Zhao et al. [63] indicate that ALS monocytes switch toward a proinflammatory M1 state in the peripheral circulation and contribute to ALS disease progression [63]. In ALS patients sera, M1 macrophages produce abundant pro-inflammatory cytokines, including IL-6, correlating with disease burden and TNF-α, that sustain disease progression rates and indicate the loss of macrophage-mediated neuroprotection and the predominant neurotoxic pro-inflammatory phenotype of ALS monocytes/macrophages, suggesting that monocytes from ALS patients represent a potential target for immunomodulatory therapy [64]. For example, in mice, the blockade of the pro-inflammatory cytokine IL-1β receptor decreases inflammation that, in turn, reduces motor neuron loss and prolongs the survival of mice [65]. It is now well-established that the breakdown of the BBB during ALS disease occurs and, consequently, peripheral immune cells could infiltrate into the CNS as demonstrated in mSOD1 ALS mice and patients [66]. In CNS, invading peripheral macrophages become ‘microglia-like’ and express several microglial-specific genes [67]. Moreover, several studies have suggested that upon entry into CNS, peripheral immune cells affect glial cells during mSOD1 disease progression by combining with inflammatory responses driven by microglia and becoming able to self-propagate and amplify the inflammation and brain injury. For example, in mSOD1/PU1 knockout mice transplanted with bone marrow from either Rag2–/– or Cd4–/– mice lacking lymphocytes and CD4+ T cells, an increased expression of microglial factors associated with neurotoxicity was observed [68], with a discrepancy in microglial activation state between early and late disease phases probably due to a temporal shift in the composition of the infiltrating immune cell population during disease progression or to the copy numbers of the SOD1 transgene present in mice [69]. Also, in mSOD1 mice, a microglial response-dependent neurotoxic state is observed in the presence of NK cells, whose depletion by using blocking antibodies shifts microglial activation toward a neuroprotective phenotype associated with an extended survival of mSOD1 mice [70]. In ALS patients, peripheral monocytes also invade the CNS. Mantovani was the first to observe in 2009 a significant reduction in monocyte (CD14+) number in the peripheral blood of ALS cases. In particular, the study by Mantovani shows significantly reduced CD4+CD25+ regulatory T (Treg) cell and monocyte (CD14+) levels in patients at a less severe stage of disease and a reduction in HLA-DR and CCR2 expression and monocytes markers of activation. Since resident microglia partially derive from circulating activated monocytes, the authors hypothesize that these cell types are recruited early toward the CNS area [71]. Moreover, peripheral monocytes are deregulated regarding subtype constitution, function, and gene expression, and CNS-invading monocytes have a protective role in improving MNs survival in the early phase of the disease, as demonstrated by using male transgenic mice (B6SJL-Tg(SOD1-G93A)1Gur/J. The application of human immunoglobulins or fusion proteins containing only the human Fc increased CNS invasion of peripheral monocytes and delayed the disease onset, corroborating the idea that peripheral monocytes have a protective role in the early phase of ALS [72].

Of note, even if the activated peripheral immune cells cannot gain access to the CNS, they could also indirectly affect CNS inflammation. Within the CNS, the dialogue is mainly between injured motor neurons and glia, but there is also a continual dialogue between peripheral and CNS compartments. Neuroprotection and neurotoxicity are overlapping responses of myeloid populations to signals initiated by motor neurons, which may vary with the intensity of the injury. Both peripheral and central compartments become readily involved as immunomodulatory signaling spreads from the periphery to CNS and from CNS back to the periphery.

A deep spatiotemporal analysis of ALS progression in SOD1G93A mice demonstrated that denervation occurs at the neuromuscular junction before the loss of the spinal MNs. This suggests that MNs degeneration may also begin at the distal axon and so proceed to retrograde [73]. Most likely, pre-symptomatically circulating activated monocytes/macrophages can continuously access neuromuscular junctions and distal axons as they are located outside of the BBB and progressively reach CNS along the length of the degenerating nerve fibers in muscles, sciatic nerves, and ventral roots [74].

Relatively little is still known about inflammation in skeletal muscle and, in particular, near NMJs, but the involvement of skeletal muscle in ALS as a potential therapeutic tag for the modulation of neuroinflammation at the NMJ level is indicated. Van Dyke reported in SOD1G93A rats an increase in inflammation markers in a disease phase-dependent manner. In particular, in the skeletal muscle of symptomatic and end-stage SOD1(G93A) rats, an increase in CD11b, CD68 microglial inflammatory marker and inflammatory cytokines IL-1β, and TNF-α, along with a strong expression of glial fibrillar acid protein and nestin near the NMJs, has been observed, suggesting a possible implication of peripheral macrophages in ALS [75]. Another study reports how an ineffective immune response can reduce the regeneration capacity of skeletal muscle. The experiments were conducted on mice expressing two different SOD1G93A mutations, one with slow progression and one with a rapid progression of the disease and were subjected to an injection of monocyte chemoattractant protein 1 (MCP1). The slowly progressing mutant favored the resolution of inflammation following treatment, thus confirming the protective role of MCP1 and providing interesting information on the progression of the disease due to the different phenotypes linked to the same mutation, and also suggesting the involvement of the peripheral component to preserve the muscular component [76].

The exact function of macrophages along the peripheral nerves is still controversial, as though they are protective or a contribution to disease progression has not been elucidated. Most likely, the macrophages exacerbate inflammation upon the enhancement of the synthesis and the consequent release of pro-inflammatory cytokines due to the increased levels of 4-hydroxy-2-nonenal (HNE) lipid peroxides as well as oxidative stress [77].

Chiot [78] suggested a novel perspective about the pivotal role played by peripheral macrophages situated along the axons of spinal MNs in helping during ALS. They demonstrated that a proper and timely modulation of peripheral macrophage polarization induces a polarization of microglia toward a more ‘neuroprotective’ state that improves motor function in ALS. In particular, they induce myeloablation in slow- and fast-progressing SOD1-mutant ALS mouse models at a pre-symptomatic age by busulfan chemotherapy. Then, they carry out the transplantation of GFP-expressing bone marrow cells into the bone marrow of mice to visualize the entry and to measure the amount of bone marrow-derived cell penetration into the CNS. The results achieved suggest that the levels of monocyte and macrophage infiltration to the spinal cord were negligible only during late symptomatic stages. Moreover, gene expression modulation in the periphery resulted in changes in the gene expression of central macrophages, switching them to a neuroprotective phenotype [78]. Previously, a series of gene modulation experiments using GFP+ bone marrow cells transplanted into the bone marrow at an early stage of the disease demonstrated that the modulation of ROS signaling promotes neuroprotection, resulting in a reduction in peripheral macrophage activation along the axon and of microglia in the CNS near spinal motor neuron cell bodies [79,80]. It is still controversial how peripheral macrophages influence the behavior of CNS microglia, how these cells communicate, and if similar changes also occur in motor cortex macrophages, which are known to play a significant role in upper motor neuron degeneration.

## 3. Extracellular Vesicles in the Brain

**Figure 1:**
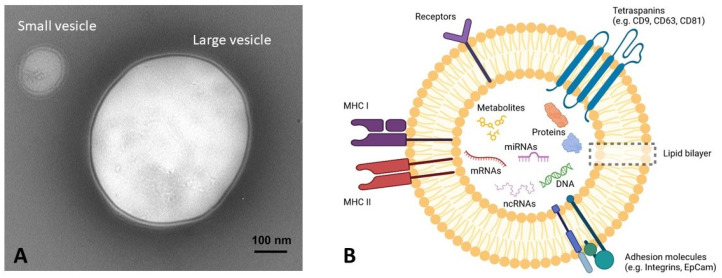
(**A**) Transmission electron microscope image of EVs released by mSOD1 NSC-34 motoneuron-like cells. (**B**) A schematic presentation of the structure of EVs. A lipid bilayer encapsulates the water-soluble cargo. Structural lipids include ceramide, sphingolipids, and phospholipids; EVs membrane proteins include major histocompatibility complex I and II (MHC I-II), cluster of differentiation, receptors, and integrins. EVs contain a variety of regulatory molecules, including DNAs, messenger RNAs (mRNA), noncoding RNAs (ncRNA), proteins, and metabolites.

EVs represent a novel route of intercellular communication with critical roles in physiological and pathological processes due to molecular cargoes of different origins (*i.e.,* nucleic acids, proteins, and metabolites) that can be delivered and transferred to both proximal and distant cells to convey specific information and modulate cellular behavior [81]. Figure 1 reports a schematic representation of molecules present in extracellular vesicles and their ultrastructure.

EVs are lipid-bound nanostructures (50–1000 nm) secreted by both normal and ill cells [82]. The International Society for Extracellular Vesicles (ISEV) recommends the use of the term “extracellular vesicles” (EVs) to generically refer to particles delimited by a lipid bilayer naturally released from the cell. The classification of EVs in subtypes is very difficult because they can be classified by considering size, biogenesis mechanism, and biochemical composition. In line with guidelines reported in Thery et al. [83], the authors are recommended to consider: (a) the physical characteristics of EVs, such as size (“small EVs” (sEVs) and “medium/large EVs” (m/lEVs), with ranges defined, for instance, respectively, <100 nm or <200 nm (small), or >200 nm (large and/or medium), or density (low, middle, high, with each range defined); (b) biochemical composition (CD63+/CD81+- EVs, annexin A5-stained EVs, etc.); or (c) the descriptions of conditions or cell of origin [83]. However, in general, EVs originating from the endosomal compartment are defined as exosomes (EXOs) regardless of the generating cells, while EVs budding from plasma membrane are known as microvesicles (MVs) or ectosomes or microparticles [84].

Based on biogenesis pathways, EVs can be primarily distinguished into two main subtypes, exosomes (EXOs, 30–200 nm) and microvesicles (MVs, up to 1000 nm) formed from the endosomal compartment or by the direct budding of the plasma membrane, respectively. The biogenesis of EXOs starts with the formation of early endosomes that accumulate intraluminal vesicles inside multivesicular bodies (MVBs) that, finally, fuse with the plasma membrane and release ILVs into the extracellular space [82,85]. Exosomes as well as microvesicles can be found in all human biofluids: urine, semen, serum, lymph, saliva, tears, nasal secretions, bile, amniotic fluid, and even breast milk [86,87,88,89,90]. A variety of bioactive molecules were found in EVs, including mRNA, microRNA, DNA, proteins, and lipids that can be delivered to recipient cells by different mechanisms, i.e., clathrin- or caveolin-mediated endocytosis, phagocytosis, micropinocytosis, and a simple fusion with the plasma membrane. The cargo can induce intracellular signaling cascades by interacting with plasma membrane receptors or directly into the cytoplasm or upon transport to the nucleus [91,92,93].

Brain activities are based on rapid and targeted communication among various cell types, including glial and neuronal cells, reshaping each other and together maintaining an optimal functioning of the brain. Glial cells, also called neuroglia, are located within the central nervous system and the peripheral nervous system and provide physical and metabolic support to neurons, including neuronal insulation and communication, and nutrient and waste transport. Glial cells, including astrocytes, microglia, and oligodendrocytes, account—depending on the mammalian species—for up to 66% of the total brain mass [94,95].

Both glial and neuronal cells release EVs. Among carried molecules, the very important ones are DNA, messenger RNA transcript (mRNA), microRNAs (miRNAs), and non-coding RNAs (ncRNA), which are able to modulate gene expression in target cells [96,97] and transcriptional regulatory proteins that trigger downstream signaling pathways in recipient cells [98]. Brain-derived EVs can be detected both in the cerebrospinal fluid (CSF) and blood as they are capable to cross the BBB from the brain to the bloodstream and vice versa via a mechanism that is still not well-known [21].

Numerous physiological and pathological processes are also regulated through EVs released by all CNS cells and the key role of EVs in the development of neuronal circuits is reported in several studies. For example, biologically active EVs containing miRNAs and proteins relevant to brain repair isolated from human-induced pluripotent stem cells (hPSC) neurons cultures incubated with human primary neurons increase cell proliferation and neuronal differentiation; moreover, EVs intranasally administrated in postnatal day 4 (P4) mice are internalized by neurons, microglia, and astrocytes in all adult rat mouse brain regions, and enhance hippocampal neurogenesis [99]. In neurons, EVs are involved in neuronal excitation in a Ca^2+^-like manner: neuron derived-EVs containing the α-amino-3-hydroxy-5-methyl-4-isoxazolepropionic acid (AMPA) receptor subunit GluR2 are involved in synaptic transmission potentially modifying the recipient cell excitability [100]. Neurons-derived EVs are implicated in retrograde signaling using the transfer of synaptotagmin4 (Stg4) to muscle cells able to induce a post-synaptic depolarization essential for activity-dependent synaptic growth [101].

Very few information is available about oligodendrocyte-derived EVs. Frühbeis demonstrated that glutamate triggers a calcium-mediated oligodendroglial exosome secretion that participates in bidirectional neuron–glia communication, contributing to neuronal integrity. The injection of oligodendroglia-derived exosomes into the mouse brain or supply of oligodendroglial exosomes in neurons cultured under oxidative stress or nutrient deprivation improves neuronal viability [102,103]. Also, EVs released from astrocytes can provide brain homeostasis as well as neurodevelopmental support through multiple processes. Astrocytes EVs contain synapsin I, an oligomannose-binding lectin, normally involved in neurite outgrowth from hippocampal neurons and a survival of cortical neurons upon hydrogen peroxide treatment or oxygen/glucose deprivation. By using dissociated hippocampal neurons obtained from neonatal C57BL/6 wild-type and NCAM-deficient mice, and EVs obtained from the cerebral cortex of newborn C57BL/6 mice astrocytes, Wang demonstrated high neuronal activity and/or oxidative stress, synapsin release from exosomes, neurite outgrowth, and neuronal survival [104]. A recent study reports that astrocytes-derived EVs contain proteins involved in promoting neuronal survival and neuroprotection. By using human astrocytic and differentiated neuronal cell lines, it has been demonstrated that the neuroprotective protein apolipoprotein D (ApoD) is exclusively transported by EVs from astrocytes to neurons. Moreover, conditioned media derived from ApoD knock-out astrocytes exert a partial protection of oxidatively stressed neurons, and a supply of EVs ApoD-positive astrocytic cell line-derived medium exerts full neuroprotection [105]. Moreover, young astrocyte-derived EVs support oligodendrocyte differentiation via the transfer of protein tyrosine phosphatase receptor type Z1 (PTPRZ), a tyrosine phosphatase involved in the differentiation of oligodendrocyte progenitor cells [106].

In pathological conditions, including neurodegenerative diseases and cancer, EVs are pivotal players in brain homeostasis. A common feature of these diseases is the elicitation of inflammation. It has been demonstrated that EVs released from activated microglia can modulate neuronal activity. Inflammatory microglia produce EVs enriched in a set of miRNAs, including miR-146a-5p, regulating the expression of key synaptic proteins. The prolonged exposure of primary neuronal cultures obtained from the hippocampi of 18-day-old fetal Sprague–Dawley rats to EVs released from activated inflammatory microglia leads to a significant decrease in dendritic spine density and a loss of excitatory synapses [107]. Astrocyte-derived ATP induces in nearby microglia the formation and the shedding of EVs containing the pro-inflammatory cytokine IL-1beta that, upon ATP stimulation, is massively released from these cells upon the rapid activation of acid sphingomyelinase, which moves to the plasma membrane outer leaflet, thus further propagating the inflammatory response [108,109]. Microglia-derived EVs also contain a set of proteins implicated in cell adhesion/extracellular matrix organization, autophagy–lysosomal pathway, and cellular metabolism, that may influence the general response occurring in the neuronal microenvironment [110]. Also in neurodegenerative diseases, such as Alzheimer’s disease (AD), Parkinson’s disease (PD), and Creutzfeldt–Jakob disease (CJD), characterized by the aggregation, deposition, and spread of specific misfolded proteins Aβ and hyperphosphorylated Tau, α-synuclein, and the pathogenic form of the prion protein (PrPSc), respectively, a shedding of EVs by neuronal and non-neuronal cells has been reported [111]. However, it is still not clear if EVs are relevant disease propagators, or rather represent a failed mechanism of clearing misfolded proteins, or whether both aspects hold true to some degree.

EVs are released by microglia/macrophages in vivo in the brain affected by multiple sclerosis (MS). The number of myeloid EVs in the CSF of both experimental autoimmune encephalomyelitis (EAE) mice, an animal model of multiple sclerosis, and MS patients in the CSF significantly increase and closely associate with the disease course. Myeloid EVs spread inflammatory signals and the treatment of EAE mice with FTY720, the first approved oral MS drug, significantly reduces the number of EVs in the CSF, suggesting that myeloid EVs could be a marker and therapeutic target of brain inflammation in MS [112]. EVs in glioma with high immunogenic potential in mice and humans have been intensively studied (as recently reviewed in [113]). EVs from patients suffering from glioblastoma (GBM) are capable to polarize CD14+ and CD163+ monocytes toward the M2 anti-inflammatory phenotype, enhancing blood serum concentrations of colony-stimulating factor 2 and 3, as well as to detect interleukin-2, -4, and -13 [114]. The ability of GBM cells to elicit an anti-inflammatory milieu has also been demonstrated also in vitro. U87MG, T98G, U373MG, and U251MG GBM cells shed a large amount of EVs both upon treatment with temozolomide and without. GBM-derived EVs, irrespective of TMZ treatment, when challenged with macrophages, modulate cell activation toward a tendentially M2b-like phenotype [115]. The induced anti-inflammatory phenotype indicates that EVs have a role in tumor growth. Finally, astrocytes release in the brain tumor microenvironment of EVs containing miR-19a are able to downregulate the tumor suppressor PTEN in tumor metastatic cells. The PTEN loss by exosomal miR-19a primes brain metastasis outgrowth [116].

## 4. Extracellular Vesicles, Immune Cells, and Amyotrophic Lateral Sclerosis: A Complex Paradigm

Communication between neurons and surrounding glial cells is a complex mechanism finely regulated by molecules expressed on the plasma membrane or released by cells according to perceived stimuli. The family of released factors includes soluble molecules directly released in the extracellular space, as well as mediators released through vesicles, which, in recent years, are taking hold also in neurodegenerative diseases both as a vehicle to spread inflammatory mediators and as a promising therapeutic way to counteract the neuronal damage. Cytokines and chemokines are the main signaling molecules able to modulate glial activation and, in turn, promote the phenotypic transformation into migratory phagocytic cells [117]. Microglia produce and secrete pro-inflammatory cytokines, including TNF-α, IL-6, and IL-1β, upon the activation of the pro-inflammatory signaling cascades, including NF-κB pathway. The release of these cytokines into the surrounding tissues creates a feed-forward loop, promoting further inflammation. In addition to cytokine signaling, recent studies have identified neuron–microglia crosstalk via fractalkine as a modality to directly modulate microglia activation [118]. Neurons constitutively express the fractalkine (CX3CL1), which inhibits microglia activation upon binding to CX3CR1 receptors located exclusively in the microglia plasma membrane [119].

Many proteins encoded by mutated genes eliciting and sustaining ALS (including *SOD1*, *TDP-43*, and *FUS*) proteins, and miRNAs involved in inflammation are present in EVs lumen. This suggested to researchers that EVs transport these molecules within the brain and shuttle them between neuronal and non-neuronal cells, contributing to disease spreading and propagation. Moreover, neuroinflammatory processes such as those observed in neurodegenerative conditions can also be transmitted both in the different regions of the brain and in different tissues. A summary of molecules, including proteins and miRNAs, able to modulate neuroinflammation in ALS by switching from pro-inflammation to anti-inflammation and vice versa, is reported in Table 2.

SOD1 has been the first ALS-associated protein retrieved in EVs. mSOD1 protein can be transferred from cell to cell via both exosome-dependent and exosome-independent routes as demonstrated by Grad and coworkers by using NSC-34 motor neuron-like cells. mSOD1 can be efficiently and repeatedly propagated from NSC-34 cells to HEK293 cell cultures via conditioned media over multiple passages and to cultured mouse primary spinal cord cells expressing wtSOD1 [145]. Moreover, the presence of mSOD1 in EVs, in particular in exosomes, released by mouse motor neuron-like NSC-34 cells overexpressing mSOD1G93A, can play a protective role against ROS production [146]. The expression of mSOD1 has a substantial impact on astrocyte protein secretion pathways by activating an unconventional secretory pathway leading to the release of exosomes that limit the formation of intracellular aggregates and overcoming mSOD1 toxicity. Astrocyte-derived exosomes transfer mSOD1 to MNs and induce their selective death with the spread of disease [147]. Also, rat primary microglial cells expressing the most-common SOD1 mutations linked to fALS (G93A and A4V) release mSOD1 through exosomes. In co-culture with primary neurons, mSOD1 transported by exosomes induces MNs cell death [148]. Finally, astrocytes- and neurons-derived EVs from non-transgenic and transgenic ALS mSOD1G93A contain pathogenic disease-causing proteins [149].

A propagation via exosomes can also occur for mTDP-43 aggregates. Feneberg [150], Ding [151], and Mackenzie [152] demonstrated the presence of exosomes enriched in TDP-43 in CSF of ALS/FTD patients, supporting the role of EVs in disease propagation and as a biomarker to assess disease progression. Moreover, EVs containing TDP-43 oligomers released by human embryonic kidney HEK-293 cells are taken up by cultured primary cortical neurons prepared from cerebral cortices of embryonic day mouse embryos (C57BL/6J). The uptake of TDP-43 oligomers causes in neurons a higher toxicity than free TDP-43 [153]. Finally, exosomes released by primary neurons obtained from the cortex of postnatal day 1 Bl/C57 mice containing TDP-43 aggregates transfer pathological protein to neuro2a cells, causing a cytoplasmic redistribution of TDP-43 and suggesting a possible contribution of secreted exosomes to the propagation of TDP-43 proteinopathy. These data are contradictory as in vivo data suggest that exosome secretion plays an overall beneficial role in the neuronal clearance of pathological TDP-43 [154].

Mutations in the *FUS* gene represent a subset of familial and sporadic ALS cases and cause an aggressive, sometimes juvenile onset disease. The mutation affects the nuclear localization signal of FUS protein, eliciting a partial mislocalization of this predominantly nuclear protein to the cytoplasm in neurons and glial cells of the spinal cord and formation of FUS-positive inclusions which exert neuronal toxicity and death, like TDP-43 and SOD1. The propagation of mutated FUS between neurons and glial cells occurs via exosomes [155].

Despite the above-reported evidence about intercellular protein transmission via EVs in ALS, their specific roles in neuromuscular pathophysiology are poorly understood. Accumulating evidence suggests that the dismantling of the neuromuscular junction caused by macrophage malfunction is an early event in the pathogenesis of ALS [156]. The modulation of macrophage activation states could stabilize the neuromuscular junction, providing protection against MNs degeneration.

As previously stated, in ALS, the degeneration of the upper and lower MNs causes a progressive paralysis of muscle cells. The involvement of exosomes in MNs degeneration has been demonstrated. In fact, a change in released exosome quality and quantity by muscle cells is toxic to MNs, which show shortened, less branched neurites, cell death, and a disrupted localization of RNA and RNA-processing proteins [157]. The contribution of exosomes in communication between lower MNS and other cells has also been demonstrated by Simeoli, suggesting an involvement of macrophages in nerve regeneration [158]. Exosomes contribute to communication between sensory neurons and macrophages after damage to the peripheral nerve. In particular, macrophages internalize sensory neuron-derived exosomes and shift toward a pro-inflammatory phenotype, becoming able to clear cellular debris and provide a suitable microenvironment for tissue repair. miR-21-5p carried by exosomes elicit the shift [159]. Furthermore, it has been shown that macrophages release exosomes that mediate ROS signaling during nerve regeneration and support the neurite outgrowth via the stimulation of PI3K-Akt signaling induced by NADPH oxidase 2 (NOX2) complexes contained in exosomes [160]. The regulation of macrophage plasticity [161] and polarization of microglia toward an anti-inflammatory phenotype [162] have been also demonstrated by using MSC-derived exosomes.

Studies report the role of monocytes in ALS both in humans and in mice [72,163]. Taking into account these results, Zondler demonstrates that peripheral CD14++ monocytes from ALS patients uptake exosomes containing TDP-43 isolated from ALS patients serum and display an impaired pro-inflammatory reaction with the secretion of the pro-inflammatory cytokines IL-6, IL-1b, and MCP-1 [164]. This can increase the amount of aggregating TDP-43 in the CNS supporting MNs damage. In fact, peripheral monocytes can infiltrate the CNS in ALS and contact TDP-43, both contained in exosomes and/or free in the microenvironment, with consequent immunological signal transmission toward immune effector cells and thereby aggravate neurodegeneration. Further, TDP-43-loaded exosomes could indirectly contribute to the misbalance of immune activation not only within the CNS, but also in the periphery as monocytes remigrate into secondary lymphatic organs after having invaded inflamed tissue.

## 5. Conclusions

The EVs/macrophages axis is a key feature of inflammatory diseases representing both a field to be explored in understanding the pathogenesis of diseases and a new hope in therapy. As the regulation of the EV/macrophage axis becomes increasingly precise, a deep understanding of this may be used as a therapeutic strategy for diseases, including neurodegenerative ones, in which the impact of inflammation strongly contributes to the pathogenesis.

Among neurodegenerative diseases, amyotrophic lateral sclerosis is a progressive and fatal motor neuron degenerative disease affecting approximately 290,000–360,000 patients worldwide with high socioeconomic significance, is hard to diagnose in the early stage, and with limited and unsatisfactory treatment. A large number of studies have shown that macrophages in a polarized state are critical to the progression of inflammation with a particular role in the regulation of the degree of inflammation. It is well-recognized that the impact of peripheral monocytes/macrophages on ALS has so far been mainly considered after the infiltration of peripheral cells into the affected CNS; in fact, as nerve macrophages were affected before microglia, the involvement of the peripheral macrophages in the disease was likely. The importance of these processes is well-established in both preclinical models and clinical data. The pro-inflammatory state of the spinal cord in ALS is mediated not only by activated microglia from the parenchyma, but also by activated inflammatory monocytes which migrated from the bloodstream into the spinal cord and brain, which release factors associated with neurodegeneration.

**Figure 2:**
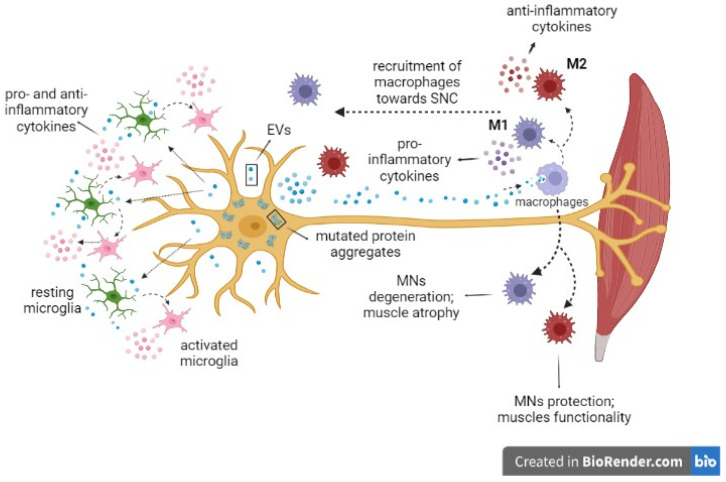
**Probable axis of macrophages–extracellular vesicles in amyotrophic lateral sclerosis.** Motor neurons (MNs) release into CNS extracellular vesicles (EVs) containing inflammatory molecules able to elicit activation of microglia that release anti- and pro-inflammatory cytokines modulating amyotrophic lateral signs. EVs pass the blood–brain barrier and reach peripheral macrophages secreting cytokines with a dual role: anti-inflammatory cytokines mediating MNs protection and ensuring muscle functionality; pro-inflammatory cytokines contributing to MNs degeneration and muscle atrophy. Peripheral macrophages can reach SNC and contribute to resolving or exacerbating neuroinflammation. Figure was drawn using the web-based tool BioRender (Toronto, Canada).

In addition to the well-accepted idea that EVs can have great application potential in diagnosis, treatment, and gene delivery in ALS, for an innovative treatment strategy, the role of EVs in recruiting macrophages into CNS or in mediating inflammatory status is becoming a very attractive field of research. In fact, EVs cargo loading offers an interesting and relatively untapped area of therapeutic target discovery. EVs play a role in disease pathogenesis through the transfer and subsequent intracellular accumulation in the target cells of both pathological proteins, such as TDP-43, SOD1, and FUS, and also inflammatory mediators, such as cytokines and chemokines, which are able to modulate the activation of resident microglia, that, in turn, can promote neuronal death. This amplifies the cellular damage carrying the pathological signs of ALS. The probable mechanism involving peripheral macrophages in ALS pathophysiology is reported in Figure 2.

Despite this, relatively little is known about these mechanisms and how they are altered in neuroinflammation. It will be very important to look to the future role of EVs and macrophages in neuroinflammation in ALS for improving the management of the disease.

## Figures and Tables

**Table 1:** Mutant genes mediating inflammatory response in ALS adapted from [26,27].

Gene	Locus	% fALS	Inheritance Pattern	Cellular/Molecular Effect	Clinical Phenotype
*C9orf72*	9p21.3	40–50%	Dominant	Microglia pro-inflammatory priming; Impaired regulation of autophagy and lysosomal pathways	Variable; Bulbar onset; Coincidence of frontotemporal dementia; Age-dependent penetrance
*OPTN*	10p13	2–3%	Dominant or recessive	Activation of the neuroinflammatory NF-kB pathway and impaired autophagy	Tendency for slower disease progression and long duration prior to respiratory dysfunction; Variable age onset
*SQSTM1*	5q35	˂1%	Dominant	Autophagy and UPS degradation; Regulator of NF-kB signaling pathway; Immune response activation	Progressive weakness by loss of motor neurons
*SOD1*	21q22	20–25%	Dominant or recessive	Major cytosolic antioxidant; Enhancement of microglial pro-inflammatory priming	Tendency for lower motor neuron predominance and lower limb onset; Cognitive changes are rare; Progression rate depends on SOD1 variant
*TBK1*	12q14	10%	Dominant	Autophagy/proteasome impairment dysregulate the mitochondria; Induction of type-1 interferon; Removal of T cells protective regulation	Encephalopathy acute; Prevalence in European populations; Variable age of onset; Different rates of progression and survival length
*TARDBP*	1p36.2	4–5%	Dominant	Enhancement of microglial pro-inflammatory priming	Earlier age of onset; Upper limb onset; Longer disease onset
*VCP*	9p13	1–2%	Dominant	Impairment of protein degradation by the ubiquitin–proteasome pathway and autophagy	Prevalence in Caucasian populations; Tendency for limb onset; Very low bulbar and respiratory onset

**Table 2:** Summary of cargo in ALS-related EVs.

Mutation	EVs Content	Biological Involvement in Inflammation	Vesicles/Sample Type	Ref.
N.A.	miRNAs		Neural enriched fraction of exosomes Peripheral human blood from ALS patients	[120]
	miR-451a	Inflammation reduction via TLR4		[121]
	miR-15-5p	Inflammation induction via NFkB and TNIP2		[122]
	miR-21-5p	Downregulation of IL-6/STAT3 pathway		[123]
	miR-16-5p	TNFα, IL-8, IL-6, IL-4 regulation		[124]
	miR-223-3p	Autophagy regulation via ATG16L1 Upregulation of IL-1β and TNFα		[125]
	miR-142-3p	Regulation of expression of IL-1β		[126]
	miR-27b-3p	Regulation of expression of IL-1β, IL-6 and TNFα		[127]
SOD1	miR-124-3p	Activation of NFkB pathway Upregulation of MMP2 and MMP9	Mice lumbar cord tissue-derived exosomes	[128,129]
TDP-43	Proteins		Motor cortex exosomes Post-mortem tissues	[130]
	CD177	Neutrophil activation		[131]
	IGHV3-43	Adaptive immunity ignition		[132]
	LBP	Innate immunity ignition via TLR		[133]
	RPS29	Modulation of NFkB signaling		[134]
	S100A9	Secretion of IL-1β, IL-6 and TNFα		[135]
	SAA1	Upregulation of inflammatory mediators (CAM, MMP, cytokines, ROS, chemokines)		[136]
	SCAMP4	Regulation of pro-inflammatory SASP		[137]
	SLC16A1	Proliferation of CD8+ T lymphocytes		[138]
N.A.	IL-6	Pro-inflammatory cytokine	Astrocytes-derived exosomes	[139]
C9orf72	BLMH	Regulation of inflammatory chemokines release	CSF-derived EVs	[140]
SOD1	CD163	Macrophages activation	Mice spinal cord Treg-derived EVs	[141]
N.D.	CHIT1	Feed-forward loop maintaining inflammation	CSF-derived EVs from patients	[142]
SOD1	IL-6, iNOS, IL-1 β, IFNγ	Pro-inflammatory mediators	Mice spinal cord Treg-derived EVs	[143]
TDP-43	TNF-R2	Activation of pro-inflammatory protein	Patients CSF-derived EVs	[144]

## Data Availability

Not Applicable.
